# Morphometry of the Great Cardiac Vein in Cadaveric Hearts of South Indian Origin

**DOI:** 10.7759/cureus.23460

**Published:** 2022-03-24

**Authors:** Vasudha Kulkarni, Geethanjali HT, Tejaswi H Lokanathan

**Affiliations:** 1 Anatomy, Dr. BR Ambedkar Medical College, Bangalore, IND; 2 Anatomy, Mandya Institute of Medical Sciences, Mandya, IND; 3 Anatomy, Adichunchanagiri Institute of Medical Sciences, Mandya, IND

**Keywords:** regression analysis, humans, dissection, coronary vessels, coronary sinus, cadaver

## Abstract

Introduction

In recent years, rapid developments in procedures like cardiac pacing, targeted drug therapy, and trans coronary venous ablation have necessitated a need for a detailed study of cardiac venous anatomy. Because the number, diameter, and course of the coronary veins vary, extensive information on the patient's specific anatomy is required for the best planning of the treatment. With this background, we planned the current research to analyze the anatomy of the great cardiac vein (GCV) in terms of length and diameter, provide a formula for calculating diameter using linear regression analysis and report the frequency of formation of the triangle of Brocq and Mouchet.

Methods

We conducted this cross-sectional study on fifty-two adult human cadaveric hearts of South Indian origin collected during dissection classes for undergraduate medical students. We measured the GCV's length and diameter and applied the linear regression analysis to derive a formula for estimating the diameter of the GCV. We also noted the frequency of formation of the triangle of Brocq and Mouchet and presented it as a percentage.

Results

The mean length and width of the GCV were 67.77 mm and 2.76 mm, respectively. The formula obtained after linear regression analysis for calculating the diameter of the GCV was: the diameter of GCV=0.0089 (length of GCV vein) ± 2.147. The triangle of Brocq and Mouchet with GCV as the base was present in 97% of the hearts.

Conclusion

The length and diameter of the GCV reported in the current study were considerably lesser than the reported findings in the literature. These findings suggest significant variations in the anatomy of the cardiac veins and call for further research on the anatomy of cardiac veins.

## Introduction

According to traditional classification, the cardiac venous system has been grouped into three component venous drainage systems [1]. The first group includes the coronary sinus (CS) and its tributaries, the anterior cardiac veins (ACV), and the Thebesian veins (TV) form the second and the third group [1]. Recent classification of cardiac veins is based on the area of venous drainage and classified into two major subgroups - greater and lesser cardiac venous systems [2]. The greater cardiac venous system includes CS and tributaries, veins that drain the right ventricle, and atrial veins. The TV account for the lesser venous system [2].

The great cardiac vein (GCV) is a tributary of the CS and is the longest cardiac vein [3]. The GCV is also the most consistently found cardiac vein [3-5]. The GCV is formed close to the heart's apex in the anterior interventricular sulcus and terminates by draining into CS [3]. Termination of the GCV is marked by the valve of Vieussens, a membranous leaflet found in 60% of the hearts [6]. The course of the GCV is divided into two segments: the first segment is the anterior interventricular segment, and the second segment is known as the basal segment [7]. The GCV drains the left atrium and ventricles, including the interventricular septum [8].

The GCV also forms part of an arteriovenous triangle of Brocq and Mouchet. The anterior interventricular artery and the left circumflex artery form the lateral boundaries, and the GCV forms the base of the triangle [9]. The relationship between the left anterior descending artery and the GCV is variable. It might be superficial, deep, right, or left to the artery [9]. The relation of the GCV in the triangle of Brocq and Mouchet is of clinical significance in cases of atherosclerotic, rigid coronary arteries which can compress on the GCV [10].

The GCV is the second most common location for aneurysms of coronary veins [11]. The aneurysm of the distal end of the GCV is also reported to interfere with coronary artery bypass surgery. The congenitally corrected transposition of coronary arteries, one of the congenital heart diseases, can also affect the GCV. The GCV is also used in various procedures like the retro infusion of cardiac veins, cardiac resynchronization therapy, and percutaneous mitral annuloplasty [12]. The anatomy of GCV assumes further clinical importance because of its reported anatomical variations [13-16].

Even with such clinical significance, studies on cardiac venous anatomy have been eclipsed by studies on arterial anatomy of the heart. Further, most of the reported studies on cardiac venous anatomy have focussed on features like origin, course, and termination. Studies reporting the length and diameter of cardiac veins, especially the GCV, are scarce. With this background, we planned the current study to measure the length and diameter of the GCV, derive a formula for estimation of diameter of the GCV, and report the incidence of formation of the triangle of Brocq and Mouchet. 

## Materials and methods

We included fifty-two adult human cadaveric hearts of South Indian origin, collected during the regular dissection classes for first-year medical students over five years. The study was exempted from institutional ethical clearance as all specimens were collected from voluntarily donated cadavers to the department of anatomy, obtained with prior written consent from the immediate kin regarding the use of the body for dissection and research purposes.

Since the collected specimens were used, we could not document the exact age and sex of the source cadaver. We included all the heart specimens with intact atria and ventricles, undissected coronary vessels, and at least one cm length of superior and inferior vena cava. Specimens with disturbed coronary vasculature were excluded from the study. 

The primary author dissected the heart specimens to observe and document the anatomy of the cardiac veins. The course of the GCV was exposed after carefully removing the epicardial fat from the sternocostal surface and around the coronary sulcus of the heart [9,17]. The GCV was traced from the apex of the heart to its termination as the CS. The length of the GCV was measured from the apex of the heart to the CS using G1 metallic wire [17]. The primary author measured the maximum diameter of the GCV at the base of the triangle of Brocq and Mouchet using a digital vernier caliper. The formation of the triangle of Brocq and Mouchet was observed and documented.

The data obtained were expressed using descriptive statistics like mean, standard deviation, and range. Linear regression analysis was used to derive a formula for estimating the diameter of the GCV. The incidence of formation of the triangle of Brocq and Mouchet was expressed as a percentage.

## Results

**Figure 1:**
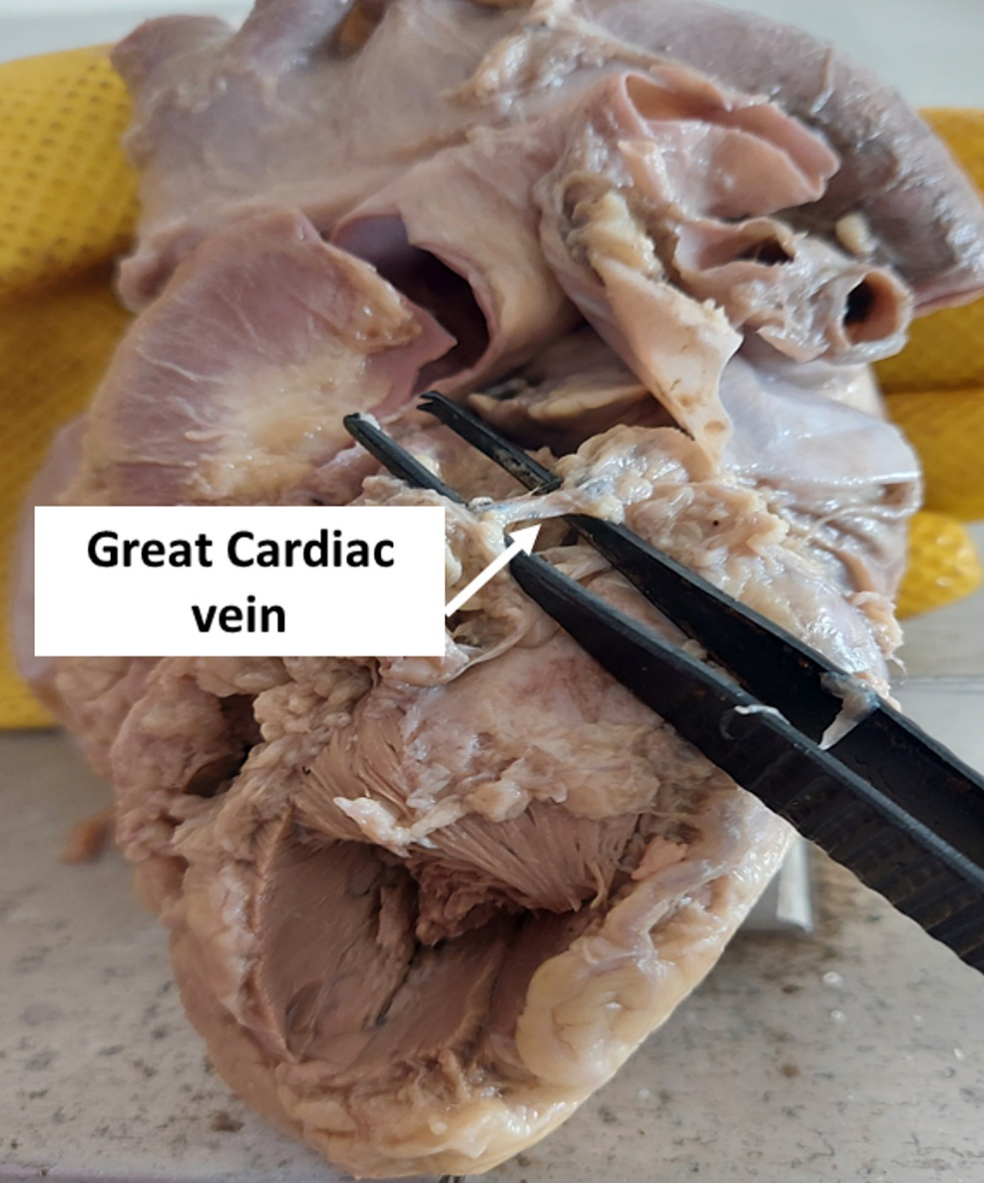
Heart specimen with a short great cardiac vein

The formation of the GCV was noted at a variable distance from the apex of the heart. The length and diameter of the GCV are listed in table 1. We observed a short GCV (Figure 1) with a length of 19 mm in one of the heart specimens included in the current study. 

**Table 1:** Measurements of the great cardiac vein

Number of hearts studied	Parameter measured	Minimum (mm)	Maximum (mm)	Range (in mm)	Mean (mm)	Standard deviation
52	Length	19	127	108	67.77	25.55
	Diameter	1	5	4	2.76	0.93

**Figure 2:**
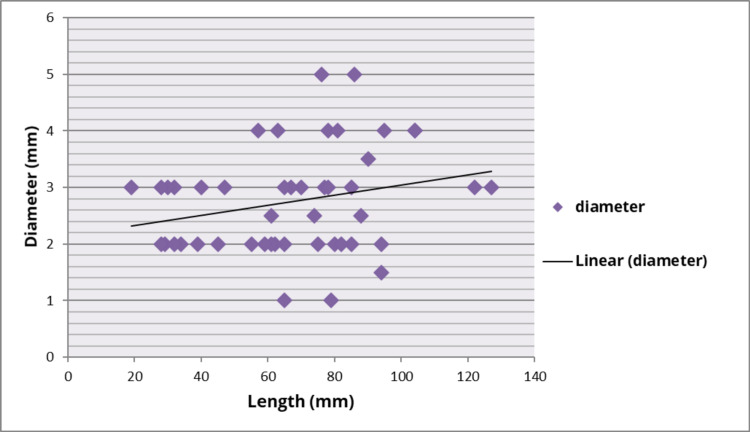
Scatter plot showing the relationship between length and diameter of the great cardiac vein

The relationship between the length and diameter of the GCV is depicted in Figure 2.

We assessed the relationship between the length and diameter of the GCV using linear regression analysis and derived the formula for calculating the diameter of the GCV. The formula obtained is: the diameter of the GCV = 0.0089 (length of the GCV) ± 2.147.

**Figure 3:**
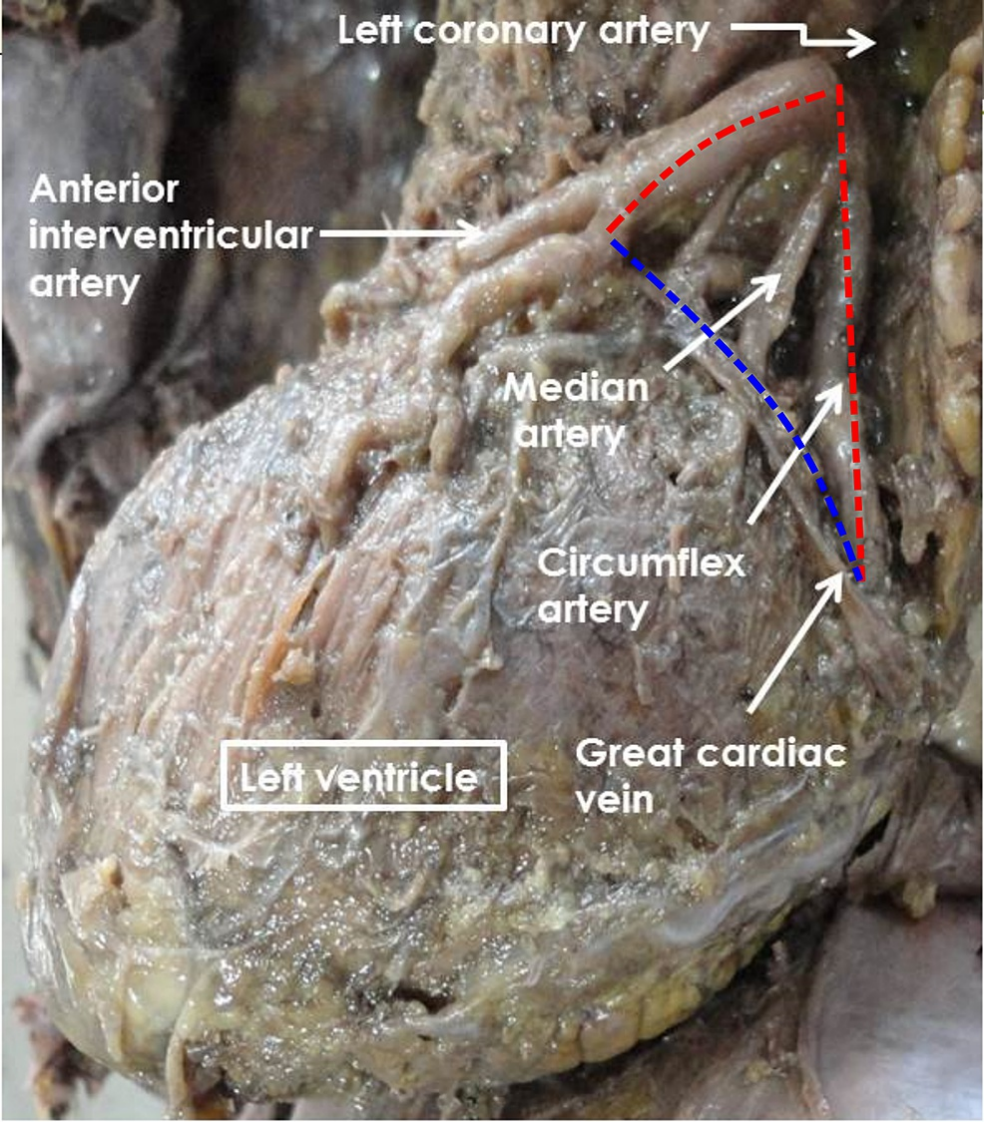
The triangle of Brocq and Mouchet Blue dotted line: GCV; short red dotted line: anterior interventricular artery; longer red dotted line: circumflex artery GCV -  great cardiac vein

We found the triangle of Brocq and Mouchet (Figure 3) with the GCV as the base in 49 hearts (97%).

## Discussion

The objective of the current study was to describe the length and diameter of the GCV, derive a formula for calculating the diameter of the GCV using linear regression analysis, and report the incidence of formation of the triangle of Brocq and Mouchet.

The mean length of the GCV in the current study was 67.77 mm. After the literature review, we could find one study that reported the length of the GCV [17]. Mehra et al. from India in 2016 reported the mean length of the GCV in 40 dissected specimens of the heart. In this study, the mean length of the GCV was 96.23±22.52 mm and ranged from 52.38 mm to 147.65 mm. The mean length of the GCV in the current study (67.77 mm) was much shorter than the study done by Mehra et al. [17]. This difference could be attributed to the source of the dissected heart specimens. The current study was done in cadaveric hearts of South Indian origin. In contrast, the study done by Mehra et al. included cadaveric hearts of North Indian origin. 

We observed a short GCV (Figure 1) with a length of 19 mm in one of the heart specimens included in the current study. In this specimen, the anterior interventricular vein ended abruptly as the intramural vein in the interventricular groove. The GCV commenced at the left and anterior part of the atrioventricular groove to terminate into the coronary artery sinus. Extensive myocardial bridging in a few heart specimens has been attributed to the variable length of the GCV. This attributes to the short length of the GCV.

The mean diameter of the GCV in the current study was 2.76 mm. According to earlier reported studies from Mehra et al. [17] and El-Maasarany et al. [18], the mean diameter of the GCV was 5.99±1.02 mm and 5.6±1.6 mm, respectively. The diameter of the GCV in the current study was much smaller than the studies done by Mehra et al. and El-Maasarany et al.. This difference in the findings could be due to the difference in the site of measurement of diameter. In the current study, the diameter was measured in the middle of the base of the triangle of the Brocq and Mouchet. In the study reported by Mehra et al. [17], the diameter was measured close to the termination of the GCV.

In the present study, we derived a formula for estimating the diameter of the GCV from its length. The coefficient value (0.0089) signifies how much the diameter of the GCV changes, given a one-unit shift in the length of the GCV. A positive coefficient in this study indicates that as the length of the GCV increases, the diameter of the GCV also tends to increase. This coefficient represents the mean increase of diameter in millimeters for every additional one millimeter in length. The length and course of the GCV are variable. The prediction of the diameter of the GCV is clinically helpful as the GCV has been reported to be the second commonest site for coronary vein aneurysms, and 1.5% of CT examinations have reported the same [12].

The triangle of Brocq and Mouchet with the GCV as the base was present in 97 % of the hearts. The reported incidence of the triangle of Brocq and Mouchet in the literature is listed in Table 2.

**Table 2:** Comparison of incidence of the triangle of Brocq and Mouchet

Authors	Year of study	Number of heart specimens studied	Place of study	Incidence of the triangle of Brocq and Mouchet
Andrade et al. [10]	2010	23	Brazil	86.9%
Bharathi et al. [19]	2013	30	South India	86.7%
Roy et al. [20]	2016	30	North India	93.3%
Suma et al. [21]	2019	104	South India	98%
Kharbuja et al. [9]	2020	30	Nepal	93.3%
Current study	2022	52	South India	97%

The formation of the triangle of Brocq and Mouchet in the current study was noted in 97% of the hearts and was similar to studies done by Suma et al. [21], Roy et al. [20], and Karbuja et al. [9]. In 2013 Bharathi et al. studied 30 cadaveric heart specimens in South India and reported the formation of the triangle of Brocq and Mouchet to be 86.9% [19]. This finding was similar to the frequency reported in the Brazilian population [10]. These findings suggest that race may have a trivial effect on the incidence of the triangle of Brocq and Mouchet.

Comparison of the present study findings with other reported morphometric studies [17,18] of the GCV suggests that cardiac veins can show variations in anatomy. In 2005 Abbara et al. performed ECG-gated multi-detector computed tomography (MDCT) in 54 patients (age, 58±7.7 [SD] years) and reported that the total length of the GCV ranged from 134 to 240 mm (mean, 182.4±23.5 mm) [22]. The mean proximal GCV diameter was 5.6±1.3 mm [22]. The knowledge of normal anatomy and variations of cardiac veins is essential before performing cardiac procedures like the retro infusion of cardiac veins, cardiac resynchronization therapy, and percutaneous mitral annuloplasty [12]. Other procedures requiring knowledge of coronary venous architecture and abnormalities include left ventricular pacing, targeted medication therapy, stem cell transfer to infarcted myocardium, and arrhythmia ablation [23-25]. Beck II procedure involves using a venous graft from descending aorta to the GCV as a conduit for oxygenated blood in myocardial ischemia [26]. These reported anatomical variations and clinical importance of the cardiac venous system further emphasize the need for a detailed assessment of the anatomy of cardiac veins in individual patients before any invasive cardiac procedures.

The strength of the current study was that it is among few of the reported studies that provide morphometric data of the GCV. The major limitations were that the specimens used for the study were formalin-fixed and not fresh cadaveric specimens. We could not document the exact age and sex of the hearts studied and trace any possible history of cardiac pathology. We wish to proceed with our research using fresh cadaveric heart specimens and correlating the findings with imaging studies.

## Conclusions

The current study reports the length and diameter of the GCV, which is scarcely reported in the literature. The length and diameter of the GCV were less compared to previously reported studies. The GCV is prone to anatomical variations in terms of length and diameter. The anatomical variations of the GCV assume clinical significance because of the need for detailed morphology and morphometry before invasive cardiac procedures. The knowledge of the anatomy of the GCV and awareness of its variations in morphology and morphometry may significantly reduce complications and improve the management of patients in need of invasive cardiac procedures.
